# Supplementary material for: HIVprotI: an integrated web based platform for prediction and design of HIV proteins inhibitors
Source: J Cheminform. 2018 Mar 9;10:12. doi: 10.1186/s13321-018-0266-y (PMC5845081; doi:10.1186/s13321-018-0266-y)
Supplement: Supplementary file 2 — Additional file 2. Source code of HIVProtI web server. [file 13321_2018_266_MOESM2_ESM.zip › HIVprotI_Source-code/hivprotI/weka-3-6-6/doc/weka/core/pmml/MappingInfo.html]

MappingInfo


---


|  |  |  |
| --- | --- | --- |
| **Overview** \| **Package** \| **Class** \| **Tree** \| **Deprecated** \| **Index** \| **Help** \| **Weka's home** | |  |
| **PREV CLASS**   **NEXT CLASS** | **FRAMES**    **NO FRAMES**     **All Classes** |  |
| SUMMARY: NESTED \| FIELD \| CONSTR \| METHOD | DETAIL: FIELD \| CONSTR \| METHOD |  |


---


## weka.core.pmml Class MappingInfo

```
java.lang.Object
  weka.core.pmml.MappingInfo
```

**All Implemented Interfaces:**: java.io.Serializable

---

``` public class MappingInfo extends java.lang.Object implements java.io.Serializable ```

Class that maintains the mapping between incoming data set structure
and that of the mining schema.

**Version:**
:   $Revision: 6464 $

**Author:**
:   Mark Hall (mhall{[at]}pentaho{[dot]}com

**See Also:**: Serialized Form

---

| **Field Summary** | |
| --- | --- |
| `static int` | `UNKNOWN_NOMINAL_VALUE`             Index for incoming nominal values that are not defined in the mining schema. |


**Constructor Summary**

`MappingInfo(Instances dataSet,
MiningSchema miningSchema,
Logger log)`


| **Method Summary** | |
| --- | --- |
| `java.lang.String` | `getFieldsMappingString()`             Get a textual description of them mapping between mining schema fields and incoming data fields. |
| `double[]` | `instanceToSchema(Instance inst, MiningSchema miningSchema)`             Convert an `Instance` to an array of values that matches the format of the mining schema. |

**Methods inherited from class java.lang.Object**

`equals, getClass, hashCode, notify, notifyAll, toString, wait, wait, wait`

**Field Detail**

### UNKNOWN\_NOMINAL\_VALUE

```
public static final int UNKNOWN_NOMINAL_VALUE
```

:   Index for incoming nominal values that are not defined in the mining
    schema.

    **See Also:**: Constant Field Values


**Constructor Detail**

### MappingInfo

```
public MappingInfo(Instances dataSet,
                   MiningSchema miningSchema,
                   Logger log)
            throws java.lang.Exception
```

**Throws:**: `java.lang.Exception`


**Method Detail**

### instanceToSchema

```
public double[] instanceToSchema(Instance inst,
                                 MiningSchema miningSchema)
                          throws java.lang.Exception
```

:   Convert an `Instance` to an array of values that matches the
    format of the mining schema. First maps raw attribute values and then
    applies rules for missing values, outliers etc.

    :   **Parameters:**: `inst` - the `Instance` to convert: `miningSchema` - the mining schema incoming instance attributes **Returns:**: an array of doubles that are values from the incoming Instances, correspond to the format of the mining schema and have had missing values, outliers etc. dealt with. **Throws:**: `java.lang.Exception` - if something goes wrong

---


### getFieldsMappingString

```
public java.lang.String getFieldsMappingString()
```

:   Get a textual description of them mapping between mining schema
    fields and incoming data fields.

    :   **Returns:**: a description of the fields mapping as a String


---


|  |  |  |
| --- | --- | --- |
| **Overview** \| **Package** \| **Class** \| **Tree** \| **Deprecated** \| **Index** \| **Help** \| **Weka's home** | |  |
| **PREV CLASS**   **NEXT CLASS** | **FRAMES**    **NO FRAMES**     **All Classes** |  |
| SUMMARY: NESTED \| FIELD \| CONSTR \| METHOD | DETAIL: FIELD \| CONSTR \| METHOD |  |


---
